# International Burden of Chronic Kidney Disease and Secondary Hyperparathyroidism: A Systematic Review of the Literature and Available Data

**DOI:** 10.1155/2015/184321

**Published:** 2015-03-31

**Authors:** Elizabeth Hedgeman, Loren Lipworth, Kimberly Lowe, Rajiv Saran, Thy Do, Jon Fryzek

**Affiliations:** ^1^EpidStat Institute, Ann Arbor, MI 48105, USA; ^2^Department of Epidemiology, University of Michigan, Ann Arbor, MI 48109, USA; ^3^School of Medicine, Vanderbilt University, Nashville, TN 37212, USA; ^4^Center for Observational Research, Amgen, Inc., Thousand Oaks, CA 91320, USA; ^5^Department of Nephrology, School of Medicine, University of Michigan, Ann Arbor, MI 48109, USA

## Abstract

The international burden of secondary hyperparathyroidism (SHPT) is unknown, but it may be estimable through the available chronic kidney disease and SHPT literature. Structured reviews of biomedical literature and online data systems were performed for selected countries to ascertain recent estimates of the incidence, prevalence, and survival of individuals with CKD and SHPT. International societies of nephrology were contacted to seek additional information regarding available data. Estimates were abstracted from 35 sources reporting estimates of CKD in 25 countries. Population prevalence estimates of CKD stages 3–5 in adults ranged from approximately 1 to 9% (China, Mexico, resp.). Estimates of the population prevalence of maintenance dialysis therapy ranged from 79 per million population (pmp; China) to 2385 pmp (Japan); incidence rates ranged from 91 pmp (United Kingdom) to 349 pmp (United States). Prevalence of SHPT among stage 5D populations was highly variable and dependent upon the disease definition used. Among the few nations reporting, approximately 30–50% of stage 5D patients had serum parathyroid hormone levels >300 pg/mL. Reported incidence and prevalence estimates across the individual nations were variable, likely reflecting differing population demographics, risk factors, etiologies, and availability of treatment through all stages of CKD.

## 1. Introduction

The increasing incidence and prevalence of chronic kidney disease (CKD), including kidney failure requiring renal replacement therapies (RRT), have drawn attention to the need for understanding accompanying mineral bone disorder (CKD-MBD). Individuals with CKD are at increased risk of bone disorders, vascular abnormalities, and premature mortality due in part to changes in calcium and phosphate homeostasis [1]. While recent guidelines focus primarily on treating renal failure populations [2, 3], work from Levin and colleagues describes early changes in mineral metabolism, particularly parathyroid hormone (PTH) concentrations, that are evident in individuals with only moderate kidney disease [4]. Thus, secondary hyperparathyroidism (SHPT), bone remodeling, and associated mineral dysfunction have been seen to begin in the setting of established CKD when individuals are either asymptomatic or unaware that they have kidney disease.

Because the increased focus on mineral and bone disorders in CKD is relatively recent, little published information is available regarding the international burden of SHPT among even renal replacement populations. Hence, understanding the total burden of SHPT may be feasible only by understanding the total burden of CKD. Nationwide registries now exist to track chronic renal failure, with additional publications providing estimates of the population burden of earlier stage disease [5, 6]. An internationally based systematic review could help estimate this burden.

In the present study we sought to systematically review and summarize the descriptive epidemiology of CKD, including SHPT, across multiple nations. Our review includes data reported by online registries, in the published literature, and through personal contact with national societies of nephrology worldwide.

## 2. Subjects and Methods

### 2.1. Disease Definition

Information on CKD stage was recorded as reported in the literature. Renal function estimates were incorporated if based on glomerular filtration rate (GFR) and albuminuria; the Cockcroft-Gault (CG), Modification of Diet in Renal Disease (MDRD), and Chronic Kidney Disease Epidemiology Collaboration (CKD-EPI) formulae were all accepted for GFR estimation [7–9]. Kidney function was classified according to the 2002 National Kidney Foundation Kidney Disease Outcomes Quality Initiative (NKF KDOQI) staging system (Table 1) as this classification was the predominant system incorporated into published reports [2]. Published statistics for later stages of disease (e.g., stages 4-5) were assumed to include only individuals not yet on maintenance renal replacement therapies, unless stated otherwise. Evidence of persistence was not required for data to be eligible for estimate inclusion.

Assessment of SHPT in maintenance dialysis populations was through reports of PTH concentration. Based on the 2002 KDOQI clinical practice guidelines, SHPT was defined as PTH > 300 pg/mL or as defined and reported by the source literature [10]. All current assays for measuring PTH were included; all reports of elevated PTH within maintenance dialysis populations were assumed due to SHPT.

### 2.2. Epidemiologic Outcomes

Incidence, prevalence, mortality, and survival statistics for populations with CKD, including RRT, and SHPT were reviewed. Statistics for CKD stages 3–5, 5D, and 5T were tabulated by stage; grouped statistics (e.g., CKD stages 3–5 and 1–5 and all RRT) were recorded as reported. Renal failure (5D, 5T) incidence and survival were variably presented as rates from day 1 or day 91 of RRT initiation; when not specified, rates were assumed to be from day 1 of RRT initiation. When both rates were available, preference was given to statistics calculated from day 91 of initiation to avoid including individuals requiring only acute/short-term replacement therapy.

### 2.3. Search Strategy, Study Selection

Epidemiologic surveillance data were reviewed for descriptive statistics pertaining to CKD and SHPT from the following regions and countries: Europe (Denmark, France, Germany, Greece, Italy, Portugal, the Russian Federation, Spain, Sweden, Netherlands, and the United Kingdom (UK)); Asia (China, India, Japan, the Republic of Korea, and Turkey); Oceania (Australia and New Zealand); and the Americas (Brazil, Canada, Mexico, and the United States (US)). These countries were selected to provide a representation of countries in multiple regions of the world for which data were readily available.

A three-stage approach to identify information on the national or regional incidence, prevalence, and survival of persons with CKD or SHPT was implemented. Searches were performed to identify renal registries making available annual data reports. Registries and societies of nephrology were identified via their affiliation with the International Society of Nephrology (ISN) [11], from online searches and personal experience of the authors. Initial searches for registries and associated data were conducted in June 2013, with checks for data updates in February 2015.

 (1) To assist with data identification, national societies of nephrology were contacted between July and August of 2013 for information and recommendations. Societies were identified via the ISN, the European Renal Association-European Dialysis and Transplant Association (ERA-EDTA) nations list, and online searches. All societies were first sent an email, with nonresponders called 2-3 times to attempt contact. Responders were informed of the review and queried in a systematic manner about national statistics for CKD and SHPT (see Supplemental Data for questionnaire in Supplementary Material available online at http://dx.doi.org/10.1155/2015/184321). Data and references shared were cross-referenced to the previous literature and renal registry searches and then included if relevant and more recent or representative than previously identified information.
 (2) Systematic literature searches were similarly performed for articles reporting national or regional statistics of CKD and SHPT; full search strings are provided in the Supplemental Data but included indexed terms such as “*population surveillance*,” “*public health surveillance*,” “*renal insufficiency, chronic*,” and “*kidney failure, chronic*.” Articles indexed in Medline between January 2000 and June 2013 were eligible for inclusion; no limitations were placed on language of publication. Articles published in languages other than English were translated with freely available online translation software [12]. Additional eligibility criteria were as follows:
    (a) study designs: observational studies only, focused on national population surveillance; preference given to studies incorporating sample weighting allowing national estimates; when national surveillance estimates not available, studies of subnational populations (e.g., regions, cities) reviewed; intervention studies excluded,
    (b) population: cohorts of all age groups, or all adults (for CKD, RRT); patients requiring renal replacement therapy (for SHPT),
    (c) outcomes: chronic kidney disease, chronic renal replacement therapy, and secondary hyperparathyroidism,
    (d) time: articles published and indexed within Medline, 2001–2013; registries reviewed regularly for updates.

Search results were scanned, with articles selected for review if their abstracts reported statistics for CKD stages 3–5, including dialysis or renal transplant, and focused on a nationally or regionally representative population. Articles were excluded from consideration if they reported data from a previously identified renal registry, if they were published in a language or alphabet not easily translated (e.g., Cyrillic), or if they had been superseded by a publication with more recent or nationally representative data. Additional information on articles and registries identified and international societies contacted can be found below in Section 3 and Tables 1–4.

### 2.4. Data Extraction, Quality Assessment

An epidemiologist familiar with the field reviewed all renal registry data; the titles, abstracts, and selected articles from the literature review; and all information obtained through contacts with the international societies and was responsible for selecting the final data and articles for inclusion. Articles were categorized by nation of surveillance and reviewed for relevant, population-representative estimates; when national estimates were unavailable, articles were reviewed for the best regionally representative estimates, regardless of survey year. Articles suggested by international society contacts were given additional weight if they met the above criteria. Population estimates from selected articles and renal registry data were extracted by trained assistants into a standard workbook. Information on the source and year(s) of the included data and the age of the included patients was obtained when available. Quality control was performed first by a second reviewer and then again by the first author, with authors reviewing half of abstracted information and all information surrounding identified errors. Final estimates presented are from a globally representative selection of nations reporting on renal surveillance. Data presented are most recent estimates, though many established renal registries have collected and published data for several decades.

## 3. Results

### 3.1. Overview

Eleven registries and/or surveillance systems were identified with freely available, online content (Table 2). Content was limited to epidemiologic surveillance of persons requiring RRT, with some sites providing additional data on PTH distributions (e.g., the Japanese Society of Dialysis and Transplantation, DOPPS). Two sites provided surveillance data of pre-RRT (not on dialysis: NOD) CKD for the US (i.e., the Centers for Disease Control and Prevention's CKD Surveillance System, the United States Renal Data System (USRDS)). Medline searches of the literature returned 4,473 CKD-related articles and 100 SHPT-related articles published between January 2000 and June 2013. Finally, 16 national societies of nephrology were successfully contacted, providing confirmation of existing (or lack of) registries and direction toward additional publications or registry information (Table 3). Ultimately, epidemiologic statistics for CKD were identified for all 21 countries, with all countries publishing some information on RRT and half publishing pre-RRT prevalence statistics (Table 4). Similarly, SHPT prevalence information among dialysis populations was identified for 13 countries.

### 3.2. Chronic Kidney Disease: Estimates Not including Renal Replacement Therapy

The literature search for recent, population-representative estimates of NOD CKD yielded 14 articles covering 13 countries; estimates for two additional countries (Australia, US) were obtained from online sites (Table 5) [13–28]. Survey sample sizes ranged from 2746 to 574,024 adults, with only one study [13] targeting individuals under the age of 18 years (*N* = 3622). Survey initiation dates ranged from 1990 to 2012.

Most estimates of adult renal function were calculated using an MDRD-based formula; one study reported results estimating function with the Cockcroft-Gault formula; [14] more recent studies reported CKD-EPI formula-based estimates either alone [15] or in combination with MDRD-based estimates [16, 17]. The lowest adult prevalence estimates of CKD stages 3–5 were from China (2009-2010 (MDRD): 1.3–2.2%), the Republic of Korea (2007–2009 (MDRD): 2.6–4.6%), and Canada (2007–2009 (CKD-EPI): 3.1%) while the highest prevalence estimates were from Japan (2005 (MDRD): 10.6%), the UK (1990–2003 (MDRD): 8.5%), Mexico (1999-2000 (CG): 8.5%), and the US (1999–2010 (MDRD): 8.0% for stages 3-4 only). Though identified through the literature review, CKD prevalence estimates from India are not reported here as estimates were only for early (i.e., stages 1–3) disease [17].

### 3.3. Chronic Kidney Disease: Estimates of Renal Replacement Therapy

Estimates of the population burden of RRT (dialysis (D) or transplant (T)) necessity were typically identified through online renal registries with publicly available content. Online information was identified for all European countries [18], the UK [19], Japan [20, 21], the Republic of Korea [22], Turkey [23], Canada [24], the US [25], Australia, and New Zealand [26]. Estimates from the Latin American Dialysis and Transplant Registry [27] and the Hong Kong Renal Registry [28] and for population-based surveys from India [29] and China [30] were identified through the published literature. At the time of this report, population estimates for the year 2012 were typically available, with estimates from the published literature being older. For some countries (e.g., Turkey, Mexico), the most recent data available were reported by a larger renal registry through personal communications [18, 25]. Estimates for Germany were available only to 2006 (personal communication, German Society of Nephrology) [31]. Countries and regions covered by an established renal registry typically reported incidence and prevalence estimates for all RRT combined, as well as prevalence estimates for dialysis alone and renal transplant alone. The Japanese Society of Dialysis and Transplant provided data only for individuals on dialysis; prevalence data of any kind were not available for India.

Incidence and prevalence statistics for RRT were reported in the unit of per million population (pmp). Contrary to the literature for earlier stage CKD, estimates for RRT included both adults and children, with the exception of data from the UK Renal Registry, which computed separate estimates for adults and children (Table 6 for dialysis only; Table 7 for all RRT). Among the European countries, unadjusted annual incidence rates (IR) and prevalence (*P*) for all RRT (Table 7) ranged from 48 to 207 pmp per year and 214 to 1670 pmp, respectively, with Portugal having the highest* P* and second highest IR. Estimates from most Asian countries were similar to those of Europe, with RRT incidence rates of 36–295 pmp and prevalence of 815–1446 pmp. Of note, the 2011 prevalence of dialysis alone in Japan was the highest estimate identified for any country, at 2385 pmp (Table 6; incidence data not reported). Within the Americas, the 2010 incidence rate of RRT in Mexico was the highest (458 pmp per year), while the 2012 prevalence of RRT in the US (1968 pmp) predominated.

Survival data were available for both the dialysis-only and all RRT populations (Tables 6 and 7), with the majority of data coverage for the dialysis-only groups. One-year survival within dialysis populations ranged from 76.0% (Mexico, 2010) to 96.1% (UK, 2011). Five-year dialysis survival was markedly lower, with the lowest reported at 36% for the US (2011).

### 3.4. Chronic Kidney Disease: Estimates of SHPT within RRT Populations

Current estimates of the global burden of SHPT within CKD populations were identified from two publications [32, 33], three contacts with international societies of nephrology (Japanese Society of Nephrology, Russian Registry of Renal Replacement Therapy, and Danish Nephrology Registry personal communication), and publicly available data from Dialysis Outcomes Practice Patterns Study (DOPPS) [34]. All sources screened patient populations requiring RRT, either* en masse* or by selecting a random sample of prevalent patients. While total population data was presumed to include children requiring dialysis, the population estimates based on random sampling focused primarily on adult patients. Parathyroid function was assessed using a PTH or intact PTH (iPTH) assay, with the threshold of SHPT typically set at PTH (or iPTH) >300 pg/mL. Across Europe and Australia, the prevalence of SHPT within dialysis populations (PTH > 300 pg/mL) ranged from 30 to 49%; prevalence within dialysis populations in the Americas (US, Canada) was estimated at 54% (Table 8). Within Asia, prevalence estimates for SHPT (iPTH > 300 pg/mL) were only identified for India (28%) and Japan (11.5%).

## 4. Discussion

The objective of this study was to provide a comprehensive evaluation and summary of the global epidemiology of CKD and associated SHPT. Because we focused on point estimates across the stages of disease (e.g., stages 3–5, 5D), we did not evaluate the annual trends in disease estimates as previous authors have [35, 36].

All countries included in this review had some type of surveillance or registry to estimate the incidence and prevalence of end stage renal disease in their population. As the collection and reporting of CKD stages 5D and 5T information have been ongoing for years in many countries, these data are the most standard and comparable. Recent, population-based estimates for more moderate stages of CKD were not available for every country. Nevertheless, it appeared that countries with higher incidence and prevalence of end stage renal disease did not always have a comparably high precursor estimate of adult CKD stages 3–5. For example, Japan's 2005 estimate of 10.6% prevalence of stages 3–5 CKD corresponded with its high ESRD incidence rate (2011: 295 pmp), while the 2012 CKD stages 3–5 prevalence estimate of 8.2% for France was accompanied by middling 5D, 5T incidence and prevalence estimate (2011 IR: 150 pmp, P: 1086 pmp). Similarly, the comparatively lower adult stages 3–5 prevalence estimate of 6.1% (2008-2009) in Portugal did not correspond with its larger 5D, 5T incidence and prevalence estimates (2011 IR: 226 pmp per year, P: 1662 pmp), which were the largest reported within Europe.

The available population CKD estimates raise questions about differences in the etiology and progression of CKD across different countries. As renal function is known to decrease normally with increasing age [37, 38], prevalence estimates may reflect different age structures within the individual countries. Similarly, differences may reflect differing population burdens of diabetes mellitus, hypertension, or polycystic kidney disease, all of which are established risk factors for CKD. Less obviously, the estimates may reflect a different propensity for cardiovascular-related mortality prior to or during end stage renal disease [39], differences in mortality risk within the first year of dialysis and longer-term survival [40], or differential availability of life-extending dialysis and transplant resources [41] or attitudes toward end-of-life palliative care. These sources of variability limit the inferences from direct comparisons across the countries and provide targets for further research.

With respect to SHPT, we observed stronger similarities reported across the dialysis-dependent populations. As most population averages of SHPT hovered between 30 and 50%, the data would initially suggest that once renal failure has occurred, the biological mechanisms underlying SHPT are only minimally influenced by population or geographic differences. Before such an assertion could be verified, more information is necessary on the rates of parathyroidectomy and drug treatment schedules across the various countries (e.g., see Lafrance et al. [42]). These data were not within the scope of our searches. Additional caution must be employed when comparing PTH concentration reported using different detection assays (e.g., PTH versus iPTH assays) as earlier generation assays detect both the full protein with calcemic activity and truncated peptides with antagonistic properties [43]. Finally, the estimates presented were likely to reflect only the prevalence of SHPT in adults, even when the total population was tested; this is due to the primary association of kidney failure with aging and long-term chronic conditions in “Western” societies. Estimates of SHPT specifically among patients under the age of 18 years may vary substantially from those presented.

The data, particularly the estimates of moderate kidney disease, may be variably comparable for a few noteworthy reasons. While we limited this work to GFR-based estimates of renal function, the literature over the past decade incorporates estimates using the Cockcroft-Gault, MDRD, and CKD-EPI based models; these equations produce estimates with reasonably similar error at eGFR < 60 mL/min/1.73 m^2^ as compared to the gold standard; [44] some nations have further adapted the equations to improve their accuracy within their populations (e.g., Japan's modified MDRD formula). Estimates are also variably comparable due to year of the survey: in some cases, the data with the best population coverage (e.g., stages 3–5 data for Mexico or Australia) were over a decade old and may no longer reflect the true population burden of disease. For example, the prevalence of both obesity and diabetes has risen sharply in Mexico, and older estimates of CKD presumably underestimate the current population burden [45].

Though single point estimates are presented here, the ongoing, cross-sectional estimations produced by the US National Health and Nutrition Examination Survey (NHANES), Korean National Health and Nutrition Examination Survey (KNHANES), and Canadian Health Measures Survey (CHMS) are worth noting because they allow review of long-term trends of early and moderate CKD prevalence within their respective populations [46–48]. In addition, population estimates based on medical history data from the UK's NEOERICA (NEw Opportunities for Early Renal Intervention by Computerized Assessment) project and Japan's annual health checks have the potential to seamlessly gather information and produce ongoing estimates without the necessity of surveillance studies [49, 50].

Despite the caveats listed above, we present these international estimates of SHPT and chronic kidney disease as a way to stimulate discussion and research. Even when renal replacement therapies are available, the estimates suggest potential differences in the incidence, progression, and/or etiology of CKD that may not be immediately explainable. As the public health communities design ways to track disease burden, the information should lead to discussion of the best practices to prevent and treat disease, which may ultimately reduce the global burden of CKD.

## Supplementary Material

Supplementary materials include the search strings used for chronic kidney disease, renal replacement therapy and secondary hyperparathyroidism searches within the Medline database. Also included is a copy of the call log and survey used for contacting the individual nephrology societies.

## Figures and Tables

**Table 1:** 2002 National Kidney Foundation Kidney Disease Outcomes Quality Initiative staging of CKD.

Stage	Definition
Stage 1	Albuminuria with eGFR ≥ 90 mL/min/1.73 m^2^
Stage 2	Albuminuria with eGFR 60–89 mL/min/1.73 m^2^
Stage 3	eGFR 30–59 mL/min/1.73 m^2^
Stage 4	eGFR 15–29 mL/min/1.73 m^2^
Stage 5	0–15 mL/min/1.73 m^2^ including dialysis (5D) and transplant (5T) recipients

**Table 2:** Registries and surveillance systems identified with online content.

Country	Registry	CKD	SHPT
Europe			
Denmark	Dansk Nefrologisk Selskab [Danish Society of Nephrology] http://www.nephrology.dk/ European Renal Association-European Dialysis and Transplant Association Registry (ERA-EDTA) http://www.era-edta-reg.org/	x	x

France	European Renal Association-European Dialysis and Transplant Association Registry (ERA-EDTA) http://www.era-edta-reg.org/ Dialysis Outcomes and Practice Patterns Study Practice Monitor (DPM) http://www.dopps.org/DPM/	x	x

Germany	Dialysis Outcomes and Practice Patterns Study Practice Monitor (DPM) http://www.dopps.org/DPM/	x	x

Greece	European Renal Association-European Dialysis and Transplant Association Registry (ERA-EDTA) http://www.era-edta-reg.org/	x	

Italy	European Renal Association-European Dialysis and Transplant Association Registry (ERA-EDTA) http://www.era-edta-reg.org/ Dialysis Outcomes and Practice Patterns Study Practice Monitor (DPM) http://www.dopps.org/DPM/	x	x

Portugal	European Renal Association-European Dialysis and Transplant Association Registry (ERA-EDTA) http://www.era-edta-reg.org/	x	

Russian Federation	European Renal Association-European Dialysis and Transplant Association Registry (ERA-EDTA) http://www.era-edta-reg.org/	x	

Spain	European Renal Association-European Dialysis and Transplant Association Registry (ERA-EDTA) http://www.era-edta-reg.org/ Dialysis Outcomes and Practice Patterns Study Practice Monitor (DPM) http://www.dopps.org/DPM/	x	x

Netherlands	European Renal Association-European Dialysis and Transplant Association Registry (ERA-EDTA) http://www.era-edta-reg.org/	x	

United Kingdom	United Kingdom Renal Registry (UKRR) http://renalreg.com/ Dialysis Outcomes and Practice Patterns Study Practice Monitor (DPM) http://www.dopps.org/DPM/	x	x

Asia			
China	—		

Hong Kong^**^	—		

India	—		

Japan	Japanese Society for Dialysis Therapy (JSDT) http://www.jsdt.or.jp/	x	x

Republic of Korea	Korean Society of Nephrology ESRD Registry http://www.ksn.or.kr/english/	x	

Turkey	European Renal Association-European Dialysis and Transplant Association Registry (ERA-EDTA) http://www.era-edta-reg.org/	x	

Oceania			
Australia (+New Zealand)	The Australia and New Zealand Dialysis and Transplant Registry (ANZDATA) http://www.anzdata.org.au/ Dialysis Outcomes and Practice Patterns Study Practice Monitor (DPM) http://www.dopps.org/DPM/	x	x

The Americas			
Brazil^*^	United States Renal Data System (USRDS) http://www.usrds.org/ ^∗∗^	x	

Canada	Canadian Organ Replacement Register (CORR) http://www.cihi.ca/ Dialysis Outcomes and Practice Patterns Study Practice Monitor (DPM) http://www.dopps.org/DPM/	x	x

Mexico^*^	United States Renal Data System (USRDS) http://www.usrds.org/	x	

USA	Centers for Disease Control and Prevention CKD Surveillance System for the United States http://apps.nccd.cdc.gov/CKD/default.aspx Dialysis Outcomes and Practice Patterns Study Practice Monitor (DPM) http://www.dopps.org/DPM United States Renal Data System (USRDS) http://www.usrds.org/	x	x

List is not to be considered comprehensive; additional data may be available elsewhere.

^*^As of 1/2014.

^**^Nation or region-specific registry data made available via publication.

**Table 3:** Societies of nephrology contacted to identify additional descriptive information on CKD.

Country	Society	Contact established?	Additional statistics/sources provided?^d^
Europe			
Denmark	Dansk Nefrologisk Selskab^a^	Yes	Yes
France	Société Francophone de Néphrologie^a^	Yes	Yes
Germany	Deutsche Gesellschaft für Nephrologie^a^	Yes	Yes
Greece	Hellenic Society of Nephrology^a^	Yes	No
Italy	Società Italiana di Nefrologia^a^	Yes	No
Portugal	Sociedade Portuguesa de Nefrologia^a^	No	—
Russian Federation	Russian Dialysis Society^a^	Yes	Yes
Spain	Sociedad Española de Nefrología^a^	No	—
Sweden	Swedish Society of Nephrology	Yes	Yes
Netherlands	Nederlandse Federatie voor Nefrologie^a^	Yes	Yes
United Kingdom	The Renal Association (UK)^a^	Yes	Yes
Asia			
China	Chinese Society of Nephrology^c^	No	
Hong Kong	—	—	—
India	Indian Society of Nephrology^b^	Yes	Yes
Japan	Japanese Society of Nephrology^b^	Yes	Yes
Republic of Korea	Korean Society of Nephrology^b^	Yes	Yes
Turkey	Turkish Society of Nephrology^a^	No	—
Americas			
Brazil	Brazilian Society of Nephrology^c^	Yes	Yes
Canada	Canadian Society of Nephrology^b^	Yes	Yes
Mexico	Mexican Institute of Nephrology Research^b^	No	—
United States	American Society of Nephrology^b^	Yes	Yes
Oceania			
Australia	Australian and New Zealand Society of Nephrology^c^	Yes	Yes

^a^Contact information obtained from ERA-EDTA national societies of nephrology list.

^b^Contact information obtained from website of country's nephrology society.

^c^Contact information obtained from ISN website.

^d^Societies often provided confirmation of existing registries or lack of existing statistics for non-RRT CKD. Some societies additionally provided references and pointers to additional online sources and/or data.

**Table 4:** Summary of identified epidemiologic data by disease and country^a^.

	CKD stages 3–5^b^			Dialysis only			All RRT			SHPT		
	I	P	S	I	P	S	I	P	S	I	P	S
Europe												
Denmark					✓	✓	✓	✓	✓		✓	
France		✓			✓		✓	✓			✓	
Germany^c^					✓		✓	✓			✓	
Greece						✓	✓	✓	✓			
Italy		✓			✓	✓	✓	✓	✓		✓	
Portugal		✓			✓		✓	✓				
Russian Federation					✓		✓	✓			✓	
Spain		✓			✓	✓	✓	✓	✓		✓	
Sweden					✓	✓	✓	✓	✓			
Netherlands					✓	✓	✓	✓	✓			
United Kingdom		✓			✓	✓	✓	✓	✓		✓	
Asia												
China		✓		✓								
Hong Kong					✓	✓		✓	✓			
India^d^				✓							✓	
Japan		✓			✓	✓					✓	
Republic of Korea		✓		✓	✓	✓	✓	✓				
Turkey		✓		✓	✓	✓	✓	✓				
Americas												
Brazil				✓	✓	✓	✓	✓			✓	
Canada		✓		✓	✓	✓	✓	✓	✓		✓	
Mexico^d^		✓			✓		✓	✓				
United States		✓		✓	✓	✓	✓	✓	✓		✓	
Oceania												
Australia		✓			✓	✓	✓	✓			✓	

I: incidence; P: prevalence; S: survival; RRT: renal replacement therapy; SHPT: secondary hyperparathyroidism.

^a^List is not to be considered comprehensive; additional data may be available elsewhere with time.

^b^Additional data available, but not shown in this writing.

^c^Information from Germany was last available in 2005.

^d^Regional estimates were included as a proxy.

**Table 5:** Prevalence of CKD stages 3–5, for Select Nations and Regions Reporting^a^.

Country	Reference	Population	*N*	Year(s) of survey	Ages surveyed (years)	Prevalence (%) stages 3–5
Europe						
Denmark	—	—	—	—	—	—
France	Bongard et al. [51]	Representative cross section of French adults, standardized to the 2009 metropolitan population in France, as part of MONA LISA study	4727	2012	35–74.9	8.2
Germany	—	—	—	—	—	—
Greece	—	—	—	—	—	—
Italy	de Nicola et al. [15]	Population representative sample of adults in Italy; preliminary data from CARHES	3559	2008	35–79	2.4 (F), 3.5 (M)^b^
Portugal	Vinhas et al. [52]	Nationally representative, random sample of adults, for the PREVADIAB study	5167	2008-2009	20–79	6.1
Russian Federation	—	—	—	—	—	—
Spain	González et al. [53]	Random selection of adults weighted to represent the Spanish population; part of the EPIRCE study	2746	2004–2008	≥20	6.8
Sweden	—	—	—	—	—	—
Netherlands	—	—	—	—	—	—
United Kingdom	Stevens et al. [49]	Adult patients with a valid SCr between Dec. 1, 1998, and Nov. 30, 2003; results age-standardized to the 2001 UK census; NEOERICA project	38,262	1990–2003	≥18	8.5
Asia						
China	Zhang et al. [54]	Multistage, stratified sample of adults from 13 provinces as part of The China National Survey of Chronic Kidney Disease	47,204	2009-2010	≥18	1.3 (M), 2.2 (F)
Hong Kong	—	—	—	—	—	—
India	—	—	—	—	—	—
Japan	Imai et al. [50]	Adults from the general population, as part of an annual health-check program; standardized to the 2005 population	574,024	2005	≥20	10.6
Republic of Korea	Kang et al. [55]	Nationally representative survey of noninstitutionalized adults for the KNHANES	15,975	2007–2009	≥20	2.6 (M), 4.6 (F)
Turkey	Süleymanlar et al. [56]	Cluster sampled survey of Turkish adults, weighted to represent the population, as part of the CREDIT study	10,748	2007-2008	≥18	5.2
	Soylemezoglu et al. [13]	Cluster sampled survey of Turkish children, weighted to represent the population, as part of the CREDIT-C study	3622	2007-2008	5–18	0.13^d^
Oceania						
Australia	AIHW [57]	Population-based survey of noninstitutionalized Australians; updated estimates from the AusDiab study	11,247	1999-2000	≥25	5.9 (M), 9.5 (F)
The Americas						
Brazil	—	—	—	—	—	—
Canada	Arora et al. [16]	CHMS: multistage, population-based survey with weighting to represent 96.3% of the Canadian population	3689	2007–2009	≥18	3.1^b^
Mexico	Amato et al. [14]	Stratified random sample of adults assigned to three primary care facilities in the city of Morelia, Mexico	3564	1999-2000	≥18	8.5^c^
United States	CDC [46]	Multistage stratified random sample of noninstitutionalized adults as part of NHANES	ns	1999–2010	≥20	8.0^e^

—: data not available.

AIHW: Australian Institute of Health and Welfare; AusDiab: Australian Diabetes, Obesity and Lifestyle Study; CARHES: Cardiovascular risk in Renal Patients of the Italian Health Examination Survey; CDC: United States Centers for Disease Control and Prevention; CHMS: Canadian Health Measures Survey; CREDIT: Chronic Renal Disease In Turkey; EPIRCE: *Estudio Epidemiológico de la Insuficiencia Renal en España*; F: female; KNHANES: Korean National Health and Nutrition Examination Survey; M: male; MONA LISA: *MONitoring NAtionaL du rISque Artériel*; NEOERICA: NEw Opportunities for Early Renal Intervention by Computerised Assessment; NHANES: National Health and Nutrition Examination Survey.

^a^Not including those on renal replacement therapies.

^b^CKD-EPI equation used for estimating renal function.

^c^Cockroft-Gault equation used for estimating renal function.

^d^Schwartz equation used for estimating renal function in children.

^e^CKD stages 3-4 only.

**Table 6:** Unadjusted incidence, prevalence, and survival of dialysis populations^a^.

Country	Reference	Source	Year(s) of survey	Ages surveyed (years)	Incidence (pmp)	Prevalence (pmp)	Survival (%)
Europe							
Denmark	ERA-EDTA [18]	Registry	2012	All	107.5^b^	461.6	1 yr (HD): 84.3^e^
France	ERA-EDTA [18]	Registry	2012	All	131.9^b^	631.3	—
Germany	Frei et al. [31]	Survey	2006	All	—	808	—
Greece	ERA-EDTA [18]	Registry	2012	All	184.3^b^	904.4	1 yr (HD): 84.3^e^
Italy	ERA-EDTA [18]	Registry	2010	All	—	756.4	1 yr (HD): 84.3^e^
Portugal	ERA-EDTA [18]	Registry	2012	All	205.5^b^	1067.9	—
Russian Federation	ERA-EDTA [18]	Registry	2011	All	—	170.0	—
Spain	ERA-EDTA [18]	Registry	2012	All	87.9–127.2^bd^	537.54	1 yr (HD): 84.3^e^
Sweden	ERA-EDTA [18]	Registry	2012	All	93.4^b^	403.0	1 yr (HD): 84.3^e^
Netherlands	ERA-EDTA [18]	Registry	2012	All	98^b^	384.3	1 yr (HD): 84.3^e^
United Kingdom	UKRR [19]	Registry	2012	≥20	91.0^b^	432.5	1 yr: 96.1
Asia							
China	Zuo et al. [30]	Survey	2008	All	—	79.1	—
India	—	—	—	—	—	—	—
Hong Kong	Ho et al. [28]	Registry	1995–2011	All	—	677.6	1 yr (HD): 83.9 1 yr (PD): 91.1 5 yr (HD): 55.7 5 yr (PD): 50.7
Japan	JSDT [20]	Survey	2011	All	—	2385.4	1 yr: 87.7
Republic of Korea	Korean ESRD Registry [35]	Registry	2013	All	200.3	1154.9	1 yr (HD): 94.9 1 yr (PD): 95.5 5 yr (HD): 73.7 5 yr (PD): 68.4
Turkey	ERA-EDTA [18]	Registry	2012	All	208.9^b^	919	5 yr (HD): 60.4^f^ 5 yr (PD): 80.6^ f^
Oceania							
Australia	ANZDATA [26]	Registry	2012	All	—	507	1 yr: 86^h^ 5 yr: 42^h^
The Americas							
Brazil	Neil et al. [5]	Survey	2011	All	149	475	5 yr (HD): 58.2^g^
Canada	CORR [24]	Registry	2012	All	150.4	682.8	1yr: 84.3 5yr: 43.8
Mexico	Rosa-Diez et al. [27]	Registry	2010	All	—	866.9	
United States	USRDS [25]	Registry	2011	All	348.8^c^	1321.3^c^	1 yr: 76.0^c^ 5 yr: 36.0^c^

—: data not available.

ANZDATA: The Australia and New Zealand Dialysis and Transplant Registry; CORR: Canadian Organ Replacement Register; ERA-EDTA: European Renal Association-European Dialysis and Transplant Association; HD: hemodialysis; JSDT: Japanese Society for Dialysis Therapy; LADTR: Latin American Dialysis and Transplant Registry; PD: peritoneal dialysis; UKRR: UK Renal Registry; USRDS: United States Renal Data System.

^a^Hemodialysis/hemofiltration and peritoneal dialysis modes.

^b^Incidence at day 91.

^c^Estimate adjusted for modality, age, gender race, ethnicity, and primary diagnosis.

^d^Estimate varies by region.

^e^Survival estimate is a combined multination estimate from ERA-EDTA; survival from day 91 forward.

^f^Estimate is from the Registry of the Nephrology, Dialysis and Transplantation in Turkey (2007).

^g^Estimate is from the Latin American Fresenius Medical Care Database.

Survival data is stratified by age; 1-year and 5-year survival information provided is for the 65–74 yr age group of Australians.

**Table 7:** Unadjusted incidence, prevalence, and survival of all renal replacement therapy populations, combined.

Country	Reference	Source	Year(s) of survey	Ages surveyed (years)	Incidence (pmp)	Prevalence (pmp)	Survival (%)
Europe							
Denmark	ERA-EDTA [18]	Registry	2012	All	115.8^a^	872.3	1 yr: 85.3^c^
France	ERA-EDTA [18]	Registry	2012	All	142.2^a^	1138.7	—
Germany	Frei et al. [31]	Registry	2006	All	213	1114	—
Greece	ERA-EDTA [18]	Registry	2012	All	185.6^a^	1135.7	1 yr: 85.3^c^
Italy	ERA-EDTA [18]	Registry	2010	All	—	905.9	1 yr: 85.3^c^
Portugal	ERA-EDTA [18]	Registry	2012	All	207.3^a^	1670.2	—
Russian Federation	ERA-EDTA [18], USRDS [25]	Registry	2012	All	48.1	213.9	—
Spain	ERA-EDTA [18]	Registry	2012	All	93.0–138.6^a^	1092.1	1 yr: 85.3^c^
Sweden	ERA-EDTA [18]	Registry	2012	All	101.8^a^	933.0	1 yr: 85.3^c^
Netherlands	ERA-EDTA [18]	Registry	2012	All	112.9^a^	923.4	1 yr: 85.3^c^
United Kingdom	UKRR [19]	Registry	2012	≥20	100.0^a^	867.1	1 yr: 87.3^a^
Asia							
China	Zuo et al. [30]	Survey	2008	All	36.1	—	—
Hong Kong	Ho et al. [28]	Registry	1995–2011	All	157	1152.5	—
India	Modi and Jha [29]	Survey	2008	All	160^b^	—	—
Japan	USRDS [25]	Registry	2011	All	295	—	—
Republic of Korea	Korean ESRD Registry [35]	Registry	2013	All	234.0	1446.4	—
Turkey	ERA-EDTA [18]	Registry	2012	All	138.6	815.6	—
Oceania							
Australia	ANZDATA [26]	Registry	2012	All	112	919	—
The Americas							
Brazil	Rosa-Diez [27]	Registry	2010	All	173.7	708.7	—
Canada	CORR [24]	Registry	2012	All	155.7	1,182.7	—
Mexico	Rosa-Diez [27]	Registry	2010	All	458.0	974.9	
United States	USRDS [25]	Registry	2012	All	358.6	1968.2	1 yr: 79.1 5yr: 41.0

—: data not available.

ANZDATA: The Australia and New Zealand Dialysis and Transplant Registry; CORR: Canadian Organ Replacement Register; ERA-EDTA: European Renal Association-European Dialysis and Transplant Association; JSDT: Japanese Society for Dialysis Therapy; LADTR: Latin American Dialysis and Transplant Registry; UKRR: UK Renal Registry; USRDS: United States Renal Data System.

^a^Incidence at day 91.

^b^Regional estimate used as proxy for national estimate.

^c^Survival estimate is a combined multination estimate from ERA-EDTA; survival from day 91 forward.

**Table 8:** Prevalence of secondary hyperparathyroidism (SHPT), where available^a^.

Country	Reference	Population	Year of survey	Ages surveyed (yrs)	SHPT definition	Prevalence (%)
Europe						
Denmark	Dansk Nefrologisk Selskabs Landsregister (DNSL) [58]	Prevalent renal replacement therapy patients	2010	All	PTH > 300 pg/mL	HD = 34 PD = 32 TX = 9
France	DOPPS, Wave 4 [34]	Randomly selected cross section of prevalent dialysis patients; weighted to represent nation	2010	≥18	PTH > 300 pg/mL	43.8
Greece	—	—	—	—	—	—
Germany	DOPPS, Wave 4 [34]	Randomly selected cross section of prevalent dialysis patients; weighted to represent nation	2010	≥18	PTH > 300 pg/mL	32.1
Italy	DOPPS, Wave 4 [34]	Randomly selected cross section of prevalent dialysis patients; weighted to represent nation	2010	≥18	PTH > 300 pg/mL	29.7
Portugal	—	—	—	—	—	—
Russian Federation	Russian Registry of Renal Replacement Therapy^a^	Prevalent hemodialysis patients	2009	All	PTH > 300 pg/mL	46.8
Spain	DOPPS, Wave 4 [34]	Randomly selected cross section of prevalent dialysis patients; weighted to represent nation	2010	≥18	PTH > 300 pg/mL	32.9
Netherlands	—	—	—	—	—	—
United Kingdom	DOPPS, Wave 4 [34]	Randomly selected cross section of prevalent dialysis patients; weighted to represent nation	2010	≥18	PTH > 300 pg/mL	42.9
Asia						
China	—	—	—	—	—	—
Hong Kong	—	—	—	—	—	—
India	Jeloka et al. [32]	Prevalent dialysis patients	NR	“Adult”	iPTH > 300 pg/mL	27.9
Japan	Japanese Society of Dialysis and Transplantation [20]	Prevalent dialysis patients	2012	All	iPTH ≥ 300 pg/mL	11.5
Republic of Korea	—	—	—	—	—	—
Turkey	—	—	—	—	—	—
Oceania						
Australia-New Zealand	DOPPS, Wave 4 [34]	Randomly selected cross section of prevalent dialysis patients; weighted to represent nation	2010	≥18	PTH > 300 pg/mL	49.1
Americas						
Brazil	Oliveira et al. [59]	Dialysis facilities across Brazil responding to a questionnaire (34% response rate representing approximately 35% of the dialysis population)	2010-2011	All	PTH > 1000 pg/mL	10.7
Canada	DOPPS, Wave 4 [34]	Randomly selected cross section of prevalent dialysis patients; weighted to represent nation	2010	≥18	PTH > 300 pg/mL	54.2
Mexico	—	—	—	—	—	—
United States	DOPPS, Wave 5 [34]	Randomly selected cross section of prevalent dialysis patients; weighted to represent nation	2012	≥18	PTH > 300 pg/mL	54

—: data not available.

DOPPS: Dialysis Outcomes and Practice Patterns Study; HD: hemodialysis; PD: peritoneal dialysis; PTH: parathyroid hormone; TX: renal transplant.

^a^By personal communication.
