# Supplementary material for: International Burden of Chronic Kidney Disease and Secondary Hyperparathyroidism: A Systematic Review of the Literature and Available Data
Source: Int J Nephrol. 2015 Mar 31;2015:184321. doi: 10.1155/2015/184321 (PMC4396737; doi:10.1155/2015/184321)
Supplement: Supplementary file 1 — Supplementary materials include the search strings used for chronic kidney disease, renal replacement therapy and secondary hyperparathyroidism searches within the Medline database. Also included is a copy of the call log and survey used for contacting the individual nephrology societies. [file 184321.f1.pdf]

INTERNATIONAL BURDEN OF CHRONIC KIDNEY DISEASE AND SECONDARY HYPERPARATHYROIDISM: A SYSTEMATIC REVIEW OF THE LITERATURE AND AVAILABLE DATA

Supplementary Material

Table of Contents

Medline Search Strings..2
International Societies of Nephrology Contact Call Questionnaire 3

Medline Search Strings

Search strings Utilized in Medline to identify relevant publications[1]

Keyword Type	Search String
Location	((((((((((((((((((((((((((((((((internationality[MeSH Terms]) OR world health[MeSH Terms]) OR population surveillance[MeSH Terms]) OR public health surveillance[MeSH Terms]) OR australia[MeSH Terms]) OR austria[MeSH Terms]) OR belgium[MeSH Terms]) OR brazil[MeSH Terms]) OR canada[MeSH Terms]) OR czech republic[MeSH Terms]) OR denmark[MeSH Terms]) OR england[MeSH Terms]) OR great britain[MeSH Terms]) OR united kingdom[MeSH Terms]) OR france[MeSH Terms]) OR germany[MeSH Terms]) OR greece[MeSH Terms]) OR india[MeSH Terms]) OR italy[MeSH Terms]) OR japan[MeSH Terms]) OR mexico[MeSH Terms]) OR northern ireland[MeSH Terms]) OR portugal[MeSH Terms]) OR russia[MeSH Terms]) OR scotland[MeSH Terms]) OR spain[MeSH Terms]) OR sweden[MeSH Terms]) OR republic of korea[MeSH Terms]) OR netherlands[MeSH Terms]) OR turkey[MeSH Terms]) OR united states[MeSH Terms]) OR wales[MeSH Terms]) OR new zealand[MeSH Terms]) OR china[MeSH Terms]) AND ("2000/01/01"[PDat] : "2013/06/21"[PDat]) AND Humans[Mesh])
Epidemiology	(((((((((((("epidemiologic monitoring"[MeSH Terms] OR "epidemiology"[MeSH Terms]) OR "prevalence"[MeSH Terms]) OR "incidence"[MeSH Terms]) OR "mortality"[MeSH Terms]) OR "survival"[MeSH Terms]) OR trends[Title/Abstract])))))) AND ("2000/01/01"[PDat] : "2013/06/21"[PDat]) AND Humans[Mesh])
Chronic Kidney Disease	(((((((((((((("kidney diseases"[MeSH Terms] OR "renal insufficiency, chronic"[MeSH Terms]) OR "kidney failure, chronic"[MeSH Terms]) OR "renal replacement therapy"[MeSH Terms]) OR chronic kidney disease[Title/Abstract]) OR ESRD[Title/Abstract]) OR end stage renal disease[Title/Abstract]) OR dialysis[Title/Abstract]) OR kidney transplant[Title/Abstract]) OR GFR[Title/Abstract]) OR eGFR[Title/Abstract]) NOT Acute[Title/Abstract]) NOT Cancer[Title/Abstract]) NOT Carcinoma[Title/Abstract]) AND ("2000/01/01"[PDAT] : "2013/06/21"[PDAT]) AND "humans"[MeSH Terms]) AND (("2000/01/01"[PDAT] : "2013/06/21"[PDAT]) AND "humans"[MeSH Terms])
Secondary Hyperparathyroidism	((((((hyperparathyroidism, secondary[MeSH Terms]) OR “secondary hyperparathyroidism”) OR SHPT))) AND ("2000/01/01"[PDat] : "2013/06/21"[PDat]) AND Humans[Mesh])

1. Search strings were composed separately by theme (e.g., location, epidemiology keywords), then grouped for the final literature search.

International Societies of Nephrology Contact Call Questionnaire

who is administering the questionnaire	who are you calling	Contact Name	phone number called	date and time	attempt #	Outcome

[Who has answered the phone/voicemail?]

[Hello, do you speak English?]

Hello, my name is ___________ and I am calling to follow up on an email that was sent to you in July. My colleagues and I are compiling international chronic kidney disease statistics and we're hoping that your Society can help us find this information.

[Voicemail: I'm sorry we missed you. We are located at EpidStat in the United States in the Eastern Time Zone. Feel free to call us at 1-734-929-9150 and ask for Elizabeth/Paula. Again, that was 1-734-929-9150. I will also try again to reach you by phone.]

If you have the time, I would like to ask you a few questions about the kidney disease statistics that your society may have publicly available.

[*If immediately referred to a different contact person, record their information below*:]

***********Questions 1 and 2 only need to be asked for Mexico, Canada, Brazil, and India*************

1. Do you know the prevalence of renal failure for your country, or do you know of a resource that tracks this information? [Prod.]

    a. Dialysis? [*If yes, complete the table and continue to #2. If no, continue with 1b and 1c*]

	Measure	Year	Source
Incidence			
Prevalence			
Mortality			
Survival			
Notes			

    b. Can you point us to a publication that has this information?

    c. Is there someone else we should talk to who would know this information?

2. We are also looking for the most recent population-based data on incidence, prevalence, mortality, and survival of **kidney transplant patients** in (COUNTRY).

    a. Do you know this information? [*If yes, complete the table and continue to #3. If no, continue with 2b and 2c*]

	Measure	Year	Source
Incidence			
Prevalence			
Mortality			
Survival			
Notes			

    b. Can you point us to a publication that has this information?

    c. Is there someone else we should talk to who would know this information?

*******************START QUESTIONNAIRE HERE FOR ALL OTHER COUNTRIES********************

3. Do you know the prevalence of chronic kidney disease for your country, or do you know of a resource that tracks this information? [Prod.]

    i. Yes

	Measure	Year	Source
Prevalence			
Incidence			
Mortality			
Survival			
Notes			

    ii. Can you point us to a publication that has this information?

    iii. Is there someone else we should talk to who would know this information?

4. [*Ask this question only if #1 indicated that data was available*]

    Do you know the prevalence of the individual stages of chronic kidney disease, or is there a resource that tracks this information?

    [*If yes, complete the tables and continue to #3, if no, go to b and c*]

    i. Stage 1

	Measure	Year	Source
Prevalence			
Incidence			
Mortality			
Survival			
Notes			

    ii. Stage 2

	Measure	Year	Source
Prevalence			
Incidence			
Mortality			
Survival			

Notes	

iii. Stage 3

	Measure	Year	Source
Prevalence			
Incidence			
Mortality			
Survival			
Notes			

iv. Stage 4

	Measure	Year	Source
Prevalence			
Incidence			
Mortality			
Survival			
Notes			

v. **Stage 5** (early)

	Measure	Year	Source
Prevalence			
Incidence			
Mortality			
Survival			
Notes			

b. Can you point us to a publication that has this information?

c. Is there someone else we should talk to who would know this information?

5. We're also interested in **secondary hyperparathyroidism**. Do you know the prevalence of secondary hyperparathyroidism, or is there a resource that tracks this information?

   a. Do you know this information? [*If yes, complete the table and i then go to #4. If no, continue with b and c*]

	Measure	Year	Source
Prevalence			
Incidence			
Mortality			
Survival			
Notes			

i. How is secondary hyperparathyroidism defined in these data?

b. Can you point us to a publication that has this information?

c. Is there someone else we should talk to who would know this information?

6. Is there data collected on **the medications used to treat mineral and bone disorder** in the kidney disease population in your country? [*If yes, proceed to5a, if no, go to 5c*]

Yes/no

7. Do you know the population-based usage or prescription rates of **phosphate binders, therapeutic vitamin D, calcium, and/or cinacalcet**?
    a. If yes, complete table:

Medication	Rate	Year	Source
Phosphate Binders			
Vitamin D, therapeutic			
Calcium			
Cinacalcet			
Notes			

b. Can you point us to a publication that has this information?

c. Is there someone else we should talk to who would know this information?

8. That was all of the information that we're looking for at this time, thank you very much for your time. If you have any other recommendations, please call me at 1-734-929-9150 or email me at [].

   Can we contact you again in the future with additional questions?

   a. [*If yes*] Is this phone number the best way to contact you? [*If yes, go to 8, if no, go to b*]
   b. Is there a better phone number or would you prefer email? What is your preferred phone/email?

9. Thank you again for your time and help. Goodbye.
